# Personalized digital extension services and agricultural performance: Evidence from smallholder farmers in India

**DOI:** 10.1371/journal.pone.0259319

**Published:** 2021-10-28

**Authors:** Pallavi Rajkhowa, Matin Qaim

**Affiliations:** 1 Center for Development Research (ZEF), University of Bonn, Bonn, Germany; 2 Institute for Food and Resource Economics, University of Bonn, Bonn, Germany; International Food Policy Research Institute, UNITED STATES

## Abstract

Productivity growth in smallholder agriculture is an important driver of rural economic development and poverty reduction. However, smallholder farmers often have limited access to information, which can be a serious constraint for increasing productivity. One potential mechanism to reduce information constraints is the public agricultural extension service, but its effectiveness has often been low in the past. Digital technologies could enhance the effectiveness of extension by reducing outreach costs and helping to better tailor the information to farmers’ individual needs and conditions. Using primary data from India, this study analyses the association between digital extension services and smallholder agricultural performance. The digital extension services that some of the farmers use provide personalized information on the types of crops to grow, the types and quantities of inputs to use, and other methods of cultivation. Problems of selection bias in the impact evaluation are reduced through propensity score matching (PSM) combined with estimates of farmers’ willingness to pay for digital extension. Results show that use of personalized digital extension services is positively and significantly associated with input intensity, production diversity, crop productivity, and crop income.

## 1. Introduction

In developing countries, productivity growth in small-farm agriculture can serve as an important driver of economic development and poverty reduction [1,2]. However, smallholder farmers typically face many challenges, such as unpredictable weather conditions, market risks, and limited access to information, technologies, and financial services. These and other constraints result in low productivity and low rates of market participation [3]. Hence, a key policy question for promoting rural development and poverty reduction is how the main information and market access constraints that smallholder farmers face can be overcome.

In most developing countries, agricultural extension services are the dominant method of public-sector support towards knowledge diffusion and innovation in the small-farm sector [4]. Traditionally, extension agents have either tried to educate farmers directly about best practices or have worked with selected “model farmers” who are then expected to act as information multipliers [5]. However, the effectiveness of traditional extension approaches has been limited, either because of too little funding and thus low outreach or information that is not sufficiently tailored to farmers’ needs [4,5]. The development and use of new digital extension approaches, which build on information and communication technologies (ICTs) such as mobile phones and internet platforms, could potentially improve the situation, but empirical evidence of actual impacts is scarce. In this article, we use data from smallholder farmers in India to analyse whether digital extension with personalized advice can help to increase innovation, productivity, and income.

There is a growing body of literature highlighting that digital technologies can lower the cost of communication significantly, thus reducing transaction costs, increasing market efficiency, and promoting economic growth and poverty reduction [6–11]. One strand of the literature focuses particularly on the role of mobile phones in agricultural development. For instance, several studies showed that the use of mobile phones can increase selling and reduce buying prices [12–14], enhance smallholder market access [15–18], and increase farm productivity and income [19–21]. Some studies also analysed the impacts of mobile phones on other dimensions of smallholder welfare, showing positive effects on nutrition and gender equality [22,23], off-farm employment [24], and migration [25]. Most of these studies focus on mobile phones as a simple communication tool. A few other studies looked at the effects of mobile phone-based financial services, such as mobile money, on-farm performance, and household welfare [26,27]. Overall, these studies suggest that mobile phones can be very beneficial for smallholder farmers.

Over the last few years–with the rise of high-speed internet connections and web-enabled smartphones–the use of ICTs has further evolved. Recent studies analysed the use of the internet and smartphones in rural areas of developing countries, generally finding positive effects on household welfare [28–30]. Various internet-based applications and technologies are being developed, which could have major implications for agricultural development. Cloud services, low-cost open-source software, and big data analytics contribute to the emergence of new, internet-based *agricultural technology platforms* (agri-tech platforms henceforth) that aggregate supply and demand by reducing the number of intermediaries and connecting farmers directly to agro-advisory services, input providers, retailers, or consumers. Rao et al. [31] reviewed such agri-tech platforms in India and categorized them into those that connect farmers to (i) extension and agro-advisory services, (ii) input suppliers, and (iii) buyers of agricultural produce. Similar platforms are also emerging in many other developing countries.

While various types of ICTs are increasingly used in agricultural extension, the literature on how these new digital extension services affect smallholder performance remains relatively thin. Several studies showed that providing farmers with general market and weather information through mobile phones, text messages, or internet applications can promote agricultural productivity and market efficiency [6,20,21,32–35]. There are also a few studies that analysed the effects of using training videos or call centres and interactive voice response services for farmers, with somewhat mixed results [19,36,37]. However, in these examples, ICTs were used primarily to improve the delivery of generic extension information. We are aware of only two published studies that analysed the effects of personalized advice (as opposed to standardized generic advice), and both these studies were carried out with farmers in Nigeria [38,39]: both studies showed that site-specific nutrient management recommendations provided through digital decision support tools help to increase crop yields. We contribute to this literature by analysing the association between the use of personalized digital extension services and smallholder agricultural performance in India. Unlike Arouna et al. [38] and Oyinbo et al. [39], who focused on nutrient management advice only, the digital application that we study involves a broader extension package, including advice for vegetable farmers on what species and varieties to grow, what quantities of different chemicals to use, and how to obtain these inputs at good quality and prices.

Digital technologies can help to better tailor different components of agricultural advice to farmers’ individual needs in multiple ways. For instance, predictive analytics and machine learning algorithms can be used to combine data on weather forecasts, soil conditions, market prices, and other aspects to develop and deliver site-specific agricultural recommendations. Theoretically, such digital extension services can affect smallholder households through several mechanisms. First, they can reduce information barriers by providing personalized advice on which types of crops to grow in what season, the appropriate types and quantities of inputs to use, and the best timing for the different operations and input applications. Second, they can connect farmers to new input markets by providing transparent information on local market prices and reputed brands and suppliers. Third, they can help improve farmers’ bargaining power by providing transparency and additional supplier options. Fourth, improved access to personalized information and new technologies and inputs can increase the levels of commercialization. These mechanisms will likely change farmers’ cropping patterns and increase their input intensities, crop yields, sales volumes, and incomes.

In this study, we use the example of a concrete digital extension application in India to analyse whether a positive association between personalized advice and farm performance can be observed. For the study, a survey of smallholder vegetable farmers was conducted in early-2019. Some of the farmers surveyed had already adopted the digital extension services, while others had not. Using propensity score matching and a variety of other statistical techniques, our results confirm positive and significant associations between the adoption of personalized digital advice and various farm performance indicators, including input intensity, production diversity, vegetable productivity, and vegetable income.

The rest of this article is organized as follows. Section 2 describes the survey region in India and concrete features of the digital extension services, followed by an explanation of the sampling strategy and the outcome variables. Section 3 discusses the econometric strategy for the impact evaluation. Section 4 presents and discusses the results, while Section 5 concludes.

## 2. Materials and methods

### 2.1 Ethics statement

This study was approved by the Research Ethics Committee of the Center for Development Research (ZEF), University of Bonn. Verbal consent was obtained from each study participant.

### 2.2 Study region and digital extension services

We focus on one large Farmer Producer Organisation (FPO) named ‘Mayurbhanj Agri Smart Farmer Producer Company Limited’ in Mayurbhanj District in the state of Odisha, eastern India. The FPO comprises around 1000 farmers growing vegetables, rice, and a few other crops. It was initiated in 2017 by the National Bank for Agriculture and Rural Development (NABARD), a public financial institution in India, and eKutir, an India-based Public Benefit Corporation. eKutir develops and establishes digital platforms to create more efficient markets for smallholder farmers in India and other parts of Asia and Africa. eKutir also developed the digital agri-tech platform ‘Farmex’ (*https*:*//farm-ex*.*io/*), which is of particular interest here. At the time of our data collection in 2019, the agri-tech platform was called ‘Blooom’. The name was changed to ‘Farmex’ in 2021.

Farmex offers vegetable farmers real-time agricultural extension services and a marketplace for vegetable seeds, fertilizers, and pesticides. In the future, the digital services shall be extended to the output market as well, but when we collected the data in early-2019 this was not yet the case. As part of the extension services, Farmex helps its users to plan season-wise cropping activities and provides information on best practices for growing specific vegetable crops. The platform also offers recommendations on the types and quantities of inputs to use and on relevant pests and diseases and how to control them. Moreover, to reduce issues with the use of counterfeit inputs, which are widespread in India, the platform makes suggestions on specific input brands and suppliers.

Due to low levels of education and widespread digital illiteracy among the FPO members, farmers do not receive the extension services on their mobile phones. Instead, when farmers want to adopt the digital extension services, they get in touch with the head of the FPO who then operates the internet-based application on the farmer’s behalf. In other words, the FPO head takes on the role of an extension agent, equipped with the digital technology that enables him/her to provide tailor-made agricultural advice and services to the FPO members.

Adoption of the digital extension services is voluntary for farmers and is currently free of charge. In this context, adoption is defined as the farmers’ active decision to subscribe to the agri-tech platform with all available services included. If a member of the FPO decides to adopt the services, the FPO head creates an individual account by entering personalized data, including farm-specific details such as location, land size, types of crops currently grown, and soil conditions. These details–together with the application’s algorithms on weather forecasts, market conditions, and optimal production decisions–are processed to provide personalized advice on crop selection, the schedule of agricultural activities, and input regimes. After every season, the FPO head enters additional data on the actual inputs used by each farmer, the yields obtained, and the prices to further improve the algorithms’ predictions and advice for future seasons. Data entry and updating are typically done during personal meetings between the FPO head and individual farmers. However, once the data are entered into the system, much of the actual advice is given through phone calls and text messages, thus lowering transaction costs.

### 2.3 Sampling strategy

Data for this study were collected through a survey conducted between January and March 2019. The selected FPO in Odisha has members in two blocks (Betnoti and Badasahi) and 26 villages. Out of all 26 villages, we randomly selected 20 villages (10 in each of the two blocks) for data collection (the remaining six villages were used for pre-testing the questionnaire). In each of the sampled villages, a household census was conducted, and all households were categorized into three groups (i) FPO vegetable farmers, (ii) non-FPO vegetable farmers, and (iii) non-FPO non-vegetable farmers. For this study, we were only interested in vegetable farmers because the digital extension services are related to vegetable cultivation. Hence, we only selected farmers from the first two groups on a random basis. The total sample includes 1105 vegetable-growing households, out of which 603 were members of the FPO and 502 were not. This distribution is proportional to the actual population proportions (S1 Table in the supporting information).

The digital extension services are accessible only to FPO members. However, as adoption for FPO members is voluntary, not all FPO members subscribed to the digital extension services. Of the 603 FPO members in our sample, around 77% (465) had adopted digital extension services by early-2019, the others had not although they would have been eligible. To analyse the association between the use of digital extension services and agricultural performance, we only include those farmers that had adopted digital extension services as part of the “treatment” group. Hence, the control group includes 640 farm households that did not adopt digital extension services irrespective of their FPO membership. It is important to mention that–beyond the digital agri-tech platform–none of the farmers in the sample villages had access to other types of formal extension services.

Relevant questions with our sampling strategy are why many of the vegetable farmers in the target villages were not members of the FPO and whether it is appropriate to include these non-members in the control group. In this context, it is important to stress that the FPO in Odisha is open to all farmers in the respective villages who grow vegetables, meaning that there are no eligibility criteria in terms of farm size or other factors. Farmers decide themselves whether they want to become FPO member. From the existing literature on farmer organisations, we know that farmers’ membership decisions typically depend on expected costs and benefits, which are influenced by various socioeconomic characteristics [40]. However, as explained above, the FPO in Odisha was only initiated in 2017 (less than two years prior to the survey), so that many farmers were still considering to join. S2 Table in the supplementary information compares various socioeconomic characteristics between FPO members and non-members in the control group, showing that both types of farms are similar. Hence, pooling FPO members and non-members in the control group seems justified. The existing farmer heterogeneity is addressed with econometric techniques, as is explained further below.

### 2.4 Data collection

Data from each randomly selected farm household were collected through personal interviews with the person responsible for farm management (mostly the household head) using a structured questionnaire. The interviews were conducted in the local language (Oriya) by trained enumerators who were supervised by the researchers. Before the actual survey, the questionnaire was pretested and adjusted by interviewing 60 households in the six non-sampled villages in the FPO area.

All agricultural data were collected for the 12 months from March 2018 to February 2019 to capture all seasons of the year. Details on crop production were asked separately for the Kharif, Rabi, and Zaid seasons. These seasonal data were summed up later to calculate annual input and output variables. As is well known, recall data collected from smallholder farmers on land sizes and yield are prone to measurement error, which can lead to bias in the statistical analysis [41]. In our context, measurement error is possible but of lesser concern than in many other smallholder studies, because we focus primarily on vegetable production, a commercial enterprise for farmers in the study region, for which written farm records and receipts are typically available. In addition to the agricultural data, information on various household characteristics, other economic activities, perceptions about digital technologies, and social networks was gathered. These data are used to control for possible confounding factors in the analysis.

### 2.5 Outcome variables

We want to analyse the association between using digital extension services and agricultural performance. Agricultural performance is evaluated in terms of crop production diversity, input use intensity, crop productivity, crop commercialization, and crop income. All outcomes are measured over 12 months covering the Kharif, Rabi, and Zaid seasons. The outcome variables are defined more specifically as:

- *Crop production diversity*: Farmers in the study area traditionally grow rice and vegetables. One of the stated objectives of the digital agri-tech platform and extension services is to help farmers diversify their production by growing more types of vegetables. We measure crop production diversity by counting the number of different crop species grown on the farm during the 12 months. The effect of the digital extension services on production diversity could be positive if farmers learn about growing new types of vegetables, but it could also be negative if they specialize in particularly lucrative species.
- *Input use intensity*: Input use intensity is measured in terms of the monetary expenditures for seeds, fertilizer, pesticides, and all inputs combined per acre of cropland. Input use intensity is of interest because the digital extension services provide specific advice on the types and quantity of inputs to use. Effects could be positive if farmers previously under-invested in inputs, but they could also be negative if farmers previously overused certain inputs or paid too high prices due to information asymmetry.
- *Crop productivity*: Productivity is measured in terms of the monetary value of the output produced per acre of land, whereby the total land cultivated by the farm is considered. We use monetary values because farmers grow many different types of crops for which physical weights are not easily comparable. Improved access to information through digital extension services is expected to result in higher crop productivity. Negative effects on productivity are not expected, as personalized advice on crop choices and input regimes will hardly lead to lower yields and revenues, unless the information provided is flawed.
- *Crop commercialization*: Crop commercialization is defined here as the share of total crop output sold during the 12 months. Many farmers keep some of their output for home consumption. We use average market prices for the particular crops produced to value home-consumed quantities. As the digital extension services are expected to increase productivity and also provide better access to market information, the effect on crop commercialization is also expected to be positive. As the extension provided only refers to crops, we focus on crop commercialization alone and do not include the livestock sector.
- *Crop income*: Annual crop income is calculated as the gross value of crop production (including the output not sold valued at average market prices) minus variable production costs (purchased seeds, fertilizer, pesticides, manure, hired labour, irrigation water, machinery, and transportation). It is expected that higher productivity and higher levels of commercialization will also lead to higher crop income. Farmers would hardly adopt and use digital extension services if these were associated with lower crop income.

## 3. Econometric approach

### 3.1 Modelling the adoption of digital extension services

Let the decision to adopt digital extension services be a dichotomous choice, such that *D*_*i*_ = 1 if household *i* adopts the digital extension services and *D*_*i*_ = 0 otherwise. Smallholders choose to adopt when the expected utility from using the services (*U*_*iD*_) is greater than the utility from not using them (*U*_*iN*_), such that *U*_*iD*_>*U*_*iN*_. The difference between the utility achieved from adopting and not adopting can be denoted by a latent variable Z*, such that Z* = [(*U*_*iD*_)−(*U*_*iN*_)]>0. Since Z* is a latent variable, it is unobservable [42]. However, it can be expressed in terms of observed variables as follows:

$$Z_{i}^{*}=\beta X_{i}+\varepsilon_{i},\;D_{i}=1\;[Z_{i}^{*}>0]$$ (1)

where *β* is a vector of parameters to be estimated, *X*_*i*_ is a vector of household, farm, and contextual characteristics, and *ε*_*i*_ is an error term that is assumed to be normally distributed.

The probability of households adopting the digital extension services can be expressed as:

$$\mathrm{Pr}{(D}_{i}=1|X_{i})=Pr{(Z}_{i}^{*}>0)=\mathrm{Pr}\left(\beta X_{i}+\varepsilon_{i}>0\right)=Pr(-\varepsilon_{i}<\beta X_{i})=F(\beta X_{i})$$ (2)

where *F* is the cumulative distribution function of −*ε*_*i*_ [42]. Depending on the assumptions regarding the functional form of *F*, probit or logit models can be used to model the determinants of digital service adoption.

### 3.2 Modelling impact

As explained, using digital extension services is expected to affect input use intensity, crop productivity, and income. Similar to Becerril and Abdulai [43] and Ogutu et al. [34], we link the adoption decision to the outcome variables by considering a simple model where a risk-neutral farmer maximizes income subject to a competitive output and input market and a single production function *Q*(*W*,*X*) that is continuous, strictly increasing, and strictly quasi-concave in a vector of variable inputs *W* and farm and household characteristics *X*. The household’s income function can be represented as:

maxY=PQ(W,X)−IW,subjecttoQ(W,X)≥Q (3)

where *Y* is crop income, *P* is the output market price, and *Q* is the expected crop output quantity. *I* is a column vector of input prices, and *W* is a vector of input quantities. Further, the crop income function can also be expressed as a function of adopting digital extension services *D*, as well as market output and input prices and farm and household characteristics:

Y=f(D,I,P,X) (4)

Thus, Eq (3) can be rewritten as:

maxY(D,I,P,X)=PQ(W,X)−IW,subjecttoQ(W,X)≥Q (5)

Now, applying Hotelling’s lemma with respect to input and output prices, output supply and input demand can be obtained by simple differentiation, such that:

$$\frac{dY}{dI}=-W=W(D,I,P,X)$$ (6)

$$\frac{dY}{dP}=Q=Q(D,I,P,X)$$ (7)

From Eqs (6) and (7), it can be observed that a farm household’s demand for inputs and the levels of crop output and income is influenced by the decision to adopt digital extension services, input, and output prices, as well as farm and household characteristics.

A common approach to estimate these relationships and the effect of digital extension services would be a set of regression models of the following type:

$$L_{i}=\alpha_{o}+\alpha_{1}D_{i}+\alpha_{2}X_{i}+\alpha_{3}C+\mu_{i}$$ (8)

where *L*_*i*_ is the outcome variable of interest, *C* is a vector of relevant controls, including input and output prices, and *μ*_*i*_ is a random error term. To evaluate how digital extension services are associated with the outcome variables, the coefficient *α*_1_ is of particular interest. However, estimating Eq (8) will likely generate biased estimates of *α*_1_ because farmers self-selected into adopting digital extension services, which may mean that *D*_*i*_ is correlated with the error term. As an alternative, we use propensity score matching (PSM) combined with several robustness checks. Further details are explained below.

### 3.3 Propensity score matching approach

A propensity score is the conditional probability of assignment to a particular treatment–adoption of digital extension services in our case–given a vector of observed covariates [44]. It can be specified as:

p(X)=Pr[D=1|X]=E[D|X]

$$p(X)=F\{h(X_{i})\}$$ (9)

where F{.} is a normal or logistic cumulative distribution function and *X* is a vector of observed covariates [43].

The PSM method builds on two assumptions, namely the conditional independence assumption (CIA), which requires that outcome variables be independent of treatment conditional on the propensity score [45], and the presence of common support, which requires that treatment participants have comparable participants in the control group in terms of their propensity scores [46]. If these two conditions hold, the PSM estimator for the average treatment effect on the treated (ATT) can be specified as the mean difference in the outcome variable *L* over the common support, weighting the comparison units by the propensity score distribution of the participants as follows [47]:

$$ATT=E[L_{i1}-L_{i0}|D=1]$$

$$ATT=E\{E[L_{i1}-L_{i0}|D_{i}=1,p(X)]\}$$

$$ATT=E_{p(X)|D=1}\{E[L_{i1}|D_{i}=1,p(X)]-E[L_{i0}|D_{i}=0,p(X)]\}$$

where *L*_*i*1_ denotes the outcome of households adopting and *L*_*i*0_ the outcome of households not adopting digital extension services. We are using the term ATT, which is very common in the PSM literature, even though we are not claiming to identify strictly causal effects. While we try to reduce issues of endogeneity to the extent possible, establishing causality is difficult with cross-section observational data.

Successful matching first requires choosing a set of covariates that satisfy CIA, as omitting important variables can lead to biased estimates [48]. We use a logit regression with a large number of covariates chosen based on economic theory and past literature to estimate the propensity scores of treatment and control group participants.

Nevertheless, it is still possible that unobserved factors such as personal motivation, risk preferences, or entrepreneurial skills affect treatment assignments such that CIA would not hold. In order to reduce the risk of biased estimates due to relevant unobserved factors, we use an approach similar to Meemken and Qaim [49]. As part of the survey, we conducted a hypothetical bidding game to elicit respondent’s willingness to pay (WTP) for an agri-tech platform that provides digital extension and improved access to input and output markets. After explaining the functioning of such an agri-tech platform, respondents were asked to quote the maximum price they would be willing to pay for a service that enables them to receive crop-related advisory information, order inputs that get delivered to their village, and find buyers for their output using a mobile application. The stated WTP is likely correlated with farmers’ motivation, preferences, and entrepreneurial skills, so that it may be a good proxy of relevant unobserved characteristics [49–51]. Hence, including this WTP as an additional covariate when estimating the propensity scores can help to reduce potential issues of unobserved heterogeneity.

After estimating the propensity scores, we match treatment and control group farmers using three different matching algorithms, namely nearest neighbour matching (NNM), radius matching (RM), and kernel-based matching (KBM). Using and comparing different matching algorithms is common as a robustness check. In NNM, each treated individual is matched with the three closest control group individuals in terms of their propensity scores. Here, matching is done with replacement, meaning that each control group individual can be used more than once as a match. For RM, a tolerance level on the maximum propensity score distance (caliper) is imposed. Here, the size of the caliper is defined as 0.2 of the standard deviation of the logit of the propensity score, as suggested by Austin [52]. KBM is a non-parametric matching estimator that uses weighted averages of all individuals in the control group to construct the counterfactual outcome [45,46]. After matching treatment and control group individuals based on their propensity scores, the ATT is calculated, as explained above.

Since we are estimating associations between digital extension services and several outcome variables, it is possible that some significant results occur simply due to chance meaning that false positives may be an issue. To address this potential issue when testing multiple hypotheses, we follow Arouna et al. [53] and present *p*-values corrected for the family-wise error rate (FWER), which is the probability of making at least one false discovery among a family of comparison, using the Bonferroni correction and Holm [54] adjusted *p*-values (see S4 Table in the supporting information). For our main results, we also present sharpened *q*-values adjusted for multiple hypotheses testing [55].

Recent research indicated that using propensity scores for matching may lead to biased impact estimates in some situations [56]. To reduce the likelihood of bias, we use several alternative methods as robustness checks. First, we re-estimate all ATTs using inverse-probability weighted regression adjustment (IPWRA) [57]. Second, we estimate the regression models in Eq (8) using ordinary least squares (OLS) and including WTP as an additional regressor. As explained, our WTP variable is likely correlated with relevant unobserved characteristics, so that including WTP can reduce issues of unobserved heterogeneity. This approach has become popular in recent impact studies, especially in situations where valid instruments are hard to identify [50,58].

Third, to analyse the magnitude of possible omitted variable bias we calculate bias-adjusted coefficients using Oster bounds [59]. Fourth, we use coarsened exact matching (CEM) developed by Iacus et al. [60,61]. Unlike PSM, CEM bounds the maximum imbalance between treated and control groups ex ante based on the chosen level of coarsening. CEM also eliminates the need to restrict data to common empirical support and bounds both the error in estimating the average treatment effect and the amount of model dependence [60]. The idea of CEM is to temporarily coarsen each variable into meaningful groups and then match households *exactly* on each of their observed characteristics. Once one-to-one exact matching is done using the coarsened data, a simple difference in means between the outcome variables in the treated and control group can be used to estimate the causal effect, or, if the match is not exact, a linear regression model can be used to control for differences [62].

## 4. Results and discussion

### 4.1 Descriptive statistics

Our analysis is carried out with a total of 1028 farm household observations for which complete data for all relevant variables are available. Descriptive statistics of all outcome and explanatory variables for the full sample are presented in S3 Table in the supplementary information. The average age of the household head is 51 years. The average educational level is around 7 years of schooling. Most of the households are headed by a male; only 7% are headed by a female. Around 82% of the households belong to socially disadvantaged groups, including Scheduled Castes (SC), Scheduled Tribes (ST), and Other Backward Classes (OBC). SC, ST, and OBC are among the most disadvantaged socioeconomic groups in India, recognized as needing special policy attention by the Indian Government. As is common in empirical analysis with micro-level data from India, we use binary variables for these groups to account for differences in socioeconomic status.

In terms of farm sizes, the average household owns around 1.3 acres of land, even though the operational holding is larger with about 4.7 acres. In other words, many households lease in land from other landowners. Based on the operational land holding, 63% of the sample farmers are classified as marginal (<2.5 acres) or small (2.5–5 acres), and 37% are classified as medium (5–10 acres) or large (>10 acres). Around 50% of the cropped area is irrigated. On average, households grow 7 different crop species. In terms of commercialization levels, households sell around 43% of their crop output and travel about 5 km on average to the closest input and output markets. S3 Table also shows that 43% of the sample households had adopted digital extension services at the time of the survey, while 57% had not. Table 1 shows the different types of information that digital extension adopters used.

**Table 1:** Types of information used by digital extension service adopters.

Type of information used	Percentage of adopters
Types of crops to grow	88%
Methods of cultivating selected crops	88%
Types of inputs to use	85%
Quantity of inputs to use	62%
Where to sell output	17%
Price to sell outputs	5%

Table 2 presents descriptive statistics, disaggregated by digital extension service adopters and non-adopters. On average, adopting households cultivate somewhat more land, have older heads that are more likely to be male and are better educated than non-adopting households. While many of the differences in Table 2 are statistically significant, adopting and non-adopting farms and households are still similar, so that comparative evaluation after controlling for observed differences seems justified. Looking at the outcome variables, which are shown in the lower part of Table 2, adopters of digital extension services have more diversified cropping patterns, are more commercialized, and have higher crop incomes than non-adopters.

**Table 2:** Socioeconomic characteristics of adopters and non-adopters of digital extension services.

	Adopters		Non-adopters		Parametric tests ^a^		Non-parametric tests^b^
	Mean	SD	Mean	SD	Difference	SE	*p*-value
Age of household head (years)	51.53	11.67	49.73	14.51	1.81**	(0.84)	0.021
Male household head (dummy)	0.96	0.20	0.92	0.27	0.04**	(0.02)	0.000
Household head owns a mobile phone (dummy)	0.76	0.43	0.70	0.46	0.06**	(0.03)	0.008
Illiterate: highest education of adult male (dummy)	0.05	0.23	0.10	0.29	-0.04**	(0.02)	0.043
Primary school: highest education of adult male (dummy)	0.21	0.41	0.27	0.45	-0.06**	(0.03)	0.017
Secondary school: highest education of adult male (dummy)	0.44	0.50	0.41	0.49	0.02	(0.03)	0.216
Bachelor or Masters: highest education of adult male (dummy)	0.29	0.46	0.19	0.39	0.11***	(0.03)	0.000
Scheduled tribe (dummy)	0.12	0.32	0.17	0.38	-0.05**	(0.02)	0.002
Scheduled caste (dummy)	0.12	0.32	0.21	0.41	-0.09***	(0.02)	0.000
Other backward classes (dummy)	0.56	0.50	0.44	0.50	0.12***	(0.03)	0.000
General caste (dummy)	0.21	0.41	0.18	0.38	0.03	(0.02)	0.309
Household size (number)	3.89	1.40	3.64	1.45	0.25***	(0.09)	0.000
Operated land (acres)	5.53	3.98	4.21	3.99	1.32***	(0.25)	0.000
Irrigation ratio (%)	53.98	35.75	48.49	38.56	5.49**	(2.36)	0.003
Livestock ownership (livestock units)	1.45	1.41	1.07	0.95	0.38***	(0.07)	0.000
Average distance to input and output market (km)	5.68	3.95	4.54	4.14	1.14***	(0.26)	0.000
Willingness to pay for digital agri-tech platform services (Rupees)	256.79	423.26	192.19	374.31	64.61***	(24.96)	0.000
Peer group ^c^	13.91	8.47	11.61	9.75	2.30***	(0.58)	0.000
Off farm income (dummy)	0.60	0.49	0.69	0.46	-0.09***	(0.03)	0.002
**Outcome variables**							
Number of crops grown	8.45	4.83	6.35	4.41	2.10***	(0.29)	0.000
Seed expenditure (1,000 Rupees/acre) ^d^	0.79	0.86	0.73	0.98	0.060	(0.06)	0.000
Fertilizer expenditure (1,000 Rupees/acre)^d^	1.80	1.35	1.73	1.62	0.07	(0.09)	0.005
Pesticides expenditure (1,000 Rupees/acre) ^d^	0.69	0.73	0.65	0.95	0.03	(0.054)	0.000
Input expenditure (1,000 Rupees/acre) ^d^	3.27	2.53	3.11	3.17	1.67	(1.83)	0.001
Crop productivity (1,000 Rs/acre) ^d^	16.03	18.78	14.41	14.10	16.22	(1.02)	0.010
Commercialization (share of farm output sold 0–1)	0.51	0.28	0.38	0.33	0.13***	(0.02)	0.000
Crop income (1,000 Rs/acre) ^d^	46.89	83.37	27.12	48.76	19.77***	(4.15)	0.000
Observations	440		588		1028		

Notes

^a^ *t-* test and prtest used for differences in means and proportions, respectively.

^b^ Mann-Whitney test and Fisher exact test used for continuous and nominal variables, respectively.

^c^ Number of households within the village from the same caste who adopted digital extension services.

^d^ These monetary variables are used in logarithmic form in the regression models.

* Significant at 10% level

** Significant at 5% level

***Significant at 1% level.

### 4.2 Factors influencing the adoption of digital extension services

Results of the logit model to explain the factors influencing digital extension service adoption are shown in Table 3. Age, education, size of the land operated, asset ownership, and several other farm and household characteristics have a positive influence on the decision to adopt digital extension services. We use education and mobile phone ownership as proxies for digital literacy. Measuring digital literacy more directly with one variable or index is not straightforward, apart from the fact that such a variable in our context might possibly be associated with reverse causality issues. Age of the household head and size of the land operated have a non-linear influence on adoption. As the age of the household head increases, initially, there is a positive effect, but this positive effect becomes smaller with further increasing age. Similarly, as the size of the land operated increases, at first, there is a positive effect on digital extension service adoption, but this positive effect gets smaller with further increasing land size. The turning points for age and the size of the land operated are 45.7 years and 11.6 acres, respectively.

**Table 3:** Logit estimates of the propensity to adopt digital extension services.

	Coefficient	Robust SE
Age of household head (years)	0.183***	(0.038)
Age squared	-0.002***	(0.000)
Male household head (dummy)	0.384	(0.329)
Household head owns a mobile phone (dummy)	0.332*	(0.191)
Primary school (dummy)	0.492*	(0.295)
Secondary school (dummy)	0.604**	(0.275)
Bachelor or Masters (dummy)	0.748**	(0.298)
Scheduled tribe (dummy)	-0.350	(0.288)
Scheduled caste (dummy)	-0.366	(0.302)
Other backward classes (dummy)	-0.189	(0.234)
Household size (number)	0.095*	(0.053)
Operated land (acres)	0.139***	(0.051)
Square of operated land (acres)	-0.006**	(0.003)
Irrigation ratio (%)	0.001	(0.002)
Livestock ownership (livestock units)	0.185**	(0.076)
Distance to input and output market (km)	0.028	(0.021)
WTP for digital agri-tech platform services (log)	0.295***	(0.093)
Peer group	0.031**	(0.013)
Off farm income (dummy)	-0.444***	(0.162)
Constant	-8.912***	(1.283)
Village dummies	Yes	
Observations	1028	
Log-likelihood	-580.957	
Pseudo R^2^	0.172	
*p*-value	0.0000	

* Significant at 10% level

** Significant at 5% level

***Significant at 1% level. Robust standard errors in parentheses.

The WTP variable, which proxies for unobserved factors such as motivation, preferences, and entrepreneurial skills, is also positively associated with the adoption of digital extension services, as one would expect. Further, the size of the social network has a positive effect: a larger number of people who adopted digital extension services from the same caste and living in the same village as the respondent is associated with a higher probability of individual adoption. In contrast, having off-farm income negatively influences the decision to adopt digital extension services (Table 3), probably because households pursuing off-farm income concentrate less on improving their farming business than households for whom agriculture is the only source of income. Off-farm income can have positive effects on farm investments, especially when access to agricultural credit is constrained [63]. However, the off-farm economy can be very diverse. For many farmers with very small landholdings, pursuing off-farm economic activities is often a simple survival mechanism rather than a conscious strategy to accumulate capital for farm upgrading [64].

In the logit model, we also control for distance to input and output markets. Market distance may be less relevant for the adoption of digital extension services that are offered in the local context but may certainly influence several of our outcome variables–such as input use and crop productivity–through various channels. Hence, it is important to control for general market access when calculating the propensity scores. As explained above, farmers do not operate the internet-based extension application themselves but through the FPO head. Hence, characteristics of the FPO head (motivation, communication skills, etc.) can also influence digital extension adoption and impacts. However, as our data were all collected in the same FPO with only one FPO head, controlling for FPO head characteristics is neither possible nor required to avoid bias.

**Fig 1:**
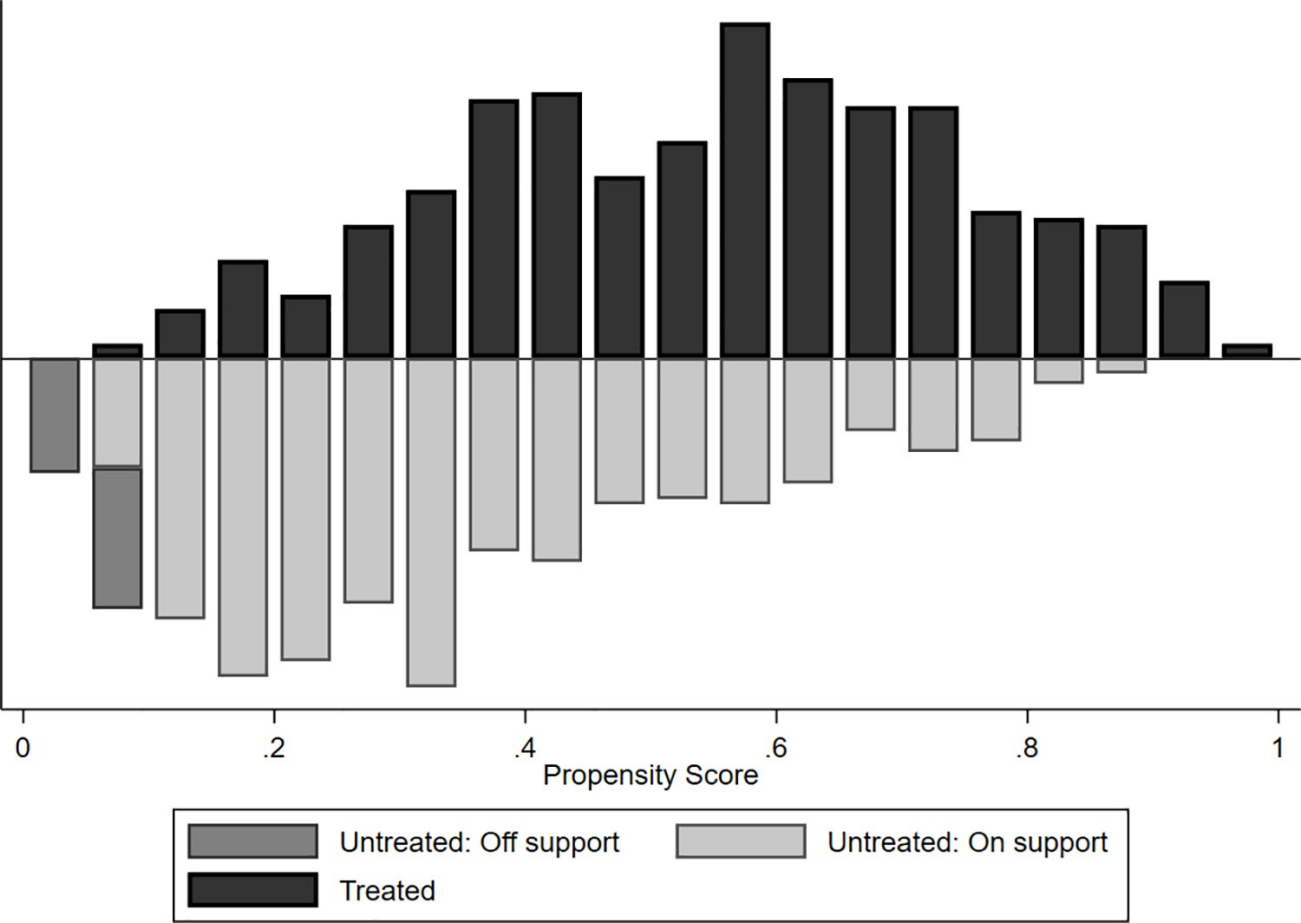
Distribution of estimated propensity scores and region of common support.

Based on these logit estimates, we calculated the propensity scores for matching treatment group households that adopted digital extension services and control group households that did not adopt. Fig 1 presents the distribution of propensity scores and the region of overlap and common support. As can be seen, there is a substantial overlap of the propensity scores of both groups, meaning that the assumption of common support is satisfied.

### 4.3 Covariate balancing tests

Table 4 presents results of the covariate balancing tests to assess how well the propensity score matching performed, or, in other words, whether digital extension service adopters and non-adopters are comparable in terms of observed covariates. For the balancing tests, we calculated mean covariate differences and conducted independent sample *t*-tests before and after matching. A match is considered successful when all *t*-tests result in non-significant differences between the treatment and the control group after matching. The results in Table 4 confirm that successful matching is achieved with all three matching algorithms.

**Table 4:** Covariate balancing tests.

	Nearest neighbour matching			Radius matching			Kernel matching		
Covariates	% Bias reduction	*p*-value mean difference, unmatched	*p*-value mean difference, matched	% Bias reduction	*p*-value mean difference, unmatched	*p*-value mean difference, matched	% Bias reduction	*p*-value mean difference, unmatched	*p*-value mean difference in, matched
Age of household head	82.2	0.035	0.727	78.0	0.035	0.633	77.1	0.035	0.621
Age squared	82.8	0.226	0.848	54.7	0.226	0.576	54.9	0.226	0.578
Male household head	82.1	0.000	0.434	96.2	0.000	0.875	99.5	0.000	0.983
Owns mobile phone	69.0	0.007	0.481	86.5	0.007	0.731	90.7	0.007	0.813
Primary school	28.2	0.016	0.160	93.9	0.016	0.891	90.1	0.016	0.825
Secondary school	89.3	0.198	0.915	88.1	0.198	0.893	84.5	0.198	0.861
Bachelor or Master	66.9	0.000	0.355	88.8	0.000	0.721	82.8	0.000	0.580
Scheduled tribe	100.0	0.002	1.000	90.6	0.002	0.750	92.6	0.002	0.801
Scheduled caste	70.0	0.000	0.290	88.7	0.000	0.644	86.7	0.000	0.588
Other backward classes	89.4	0.000	0.710	99.8	0.000	0.994	97.2	0.000	0.912
Household size	66.7	0.001	0.421	93.2	0.001	0.849	89.4	0.001	0.765
Operated land	71.3	0.000	0.188	97.8	0.000	0.910	94.5	0.000	0.775
Square of operated land	35.3	0.005	0.135	90.1	0.005	0.791	86.4	0.005	0.717
Irrigation ratio	63.0	0.021	0.483	80.9	0.021	0.686	76.0	0.021	0.612
Livestock ownership	72.5	0.000	0.180	90.5	0.000	0.588	94.8	0.000	0.767
Distance input/ output market	69.3	0.000	0.281	96.8	0.000	0.899	89.9	0.000	0.686
WTP (log)	67.2	0.000	0.128	94.6	0.000	0.767	89.6	0.000	0.569
Peer group	85.3	0.000	0.603	84.6	0.000	0.528	88.8	0.000	0.646
Off-farm income	44.3	0.002	0.202	96.0	0.002	0.915	98.5	0.002	0.967
Mean bias before matching		16.7			16.7			16.7	
Mean bias after matching		5.3			2.2			2.5	
*p*-value of LRChi^2^ unmatched		0.000			0.000			0.000	
*p*-value of LRChi^2^ matched		0.954			1.000			1.000	
Pseudo-R^2^ unmatched		0.174			0.174			0.174	
Pseudo-R^2^ matched		0.027			0.005			0.006	

Another way of testing covariate balancing is through the standardized mean bias before and after matching. These results are presented in the lower part of Table 4. As can be seen, significant mean bias reduction is achieved with all three matching algorithms. A third diagnostic test is a comparison of the pseudo-R^2^ from the logit model before and after matching, which is also shown in the lower part of Table 4. For all three matching algorithms, the pseudo-R^2^ after matching is low, suggesting that the systematic differences in the covariates that existed before matching were successfully removed.

### 4.4 Association between digital extension services and agricultural performance

Table 5 presents the PSM treatment effects, showing how the adoption of digital extension services is associated with agricultural performance. All three matching estimators indicate that adopting digital extension services is positively associated with all indicators of agricultural performance. After controlling for confounding factors, digital extension adopters cultivate one more crop species on their farm than non-adopters. The monetary outcome variables are log-transformed, so the respective ATTs can be interpreted in percentage terms. The results in Table 5 suggest that digital extension adopters have 15–20% higher input intensity, around 18% higher crop productivity, and 25–29% higher crop incomes than non-adopters. Adopters are also significantly more commercialized (by 5–7 percentage points) than non-adopters. These results remain statistically significant also after correcting for multiple hypothesis testing (Table 5).

**Table 5:** Relationship between adoption of digital extension services and agricultural performance (PSM results).

	Nearest neighbour matching		Radius matching		Kernel matching	
Outcome variable	ATT	SE	ATT	SE	ATT	SE
Number of crops grown	1.211***	(0.443)	1.017***	(0.371)	1.095***	(0.355)
	[0.024]		[0.019]		[0.006]	
Seed expenditure per acre (log)	0.170	(0.115)	0.200**	(0.099)	0.198**	(0.097)
	[0.153]		[0.046]		[0.035]	
Fertilizer expenditure per acre (log)	0.161**	(0.077)	0.149**	(0.062)	0.153**	(0.064)
	[0.039]		[0.032]		[0.028]	
Pesticide expenditure per acre (log)	0.199**	(0.102)	0.195**	(0.086)	0.198**	(0.083)
	[0.047]		[0.032]		[0.028]	
Total expenditure per acre (log)	0.188**	(0.079)	0.194***	(0.066)	0.197***	(0.066)
	[0.028]		[0.012]		[0.006]	
Crop productivity (log)	0.175**	(0.065)	0.175***	(0.059)	0.177***	(0.058)
	[0.024]		[0.032]		[0.006]	
Crop commercialization	0.074***	(0.028)	0.049**	(0.024)	0.049**	(0.023)
	[0.024]		[0.046]		[0.035]	
Crop income (log)	0.285**	(0.132)	0.254**	(0.107)	0.265**	(0.107)
	[0.039]		[0.046]		[0.024]	

ATT: average treatment effect on the treated. PSM: propensity score matching. Bootstrapped standard errors with 1,000 replications are shown in parentheses.

* Significant at 10% level

** Significant at 5% level

***Significant at 1% level. Multiple hypotheses corrected sharpened *q*-values following Anderson [54] are presented in square brackets. Unadjusted *p*-values and Bonferroni and Holm adjusted *p*-values are shown in S4 Table in the supplementary information. Results with bootstrapped standard errors and 10,000 replications are shown in S5 Table.

Several of the variables used to calculate the propensity scores may potentially be endogenous. This is particularly true for mobile phone ownership, off-farm income, and the peer group variable. Using endogenous variables for propensity score calculations is unproblematic if these are not affected by the “treatment” [45,65]. However, as reverse causality cannot be ruled out with certainty, we tested how the ATT estimates change when we exclude these potentially endogenous variables in the logit regression used to calculate the propensity scores. The ATTs are very similar to the original ones (see S6 Table in the supporting information), thus underlining the robustness of the estimates. Another variable that may potentially suffer from reverse causality is WTP, our proxy of potentially relevant unobserved heterogeneity. We also tried excluding WTP along with the other potentially endogenous variables, with results shown in S7 Table in the supplementary information. These results are slightly different from the previous estimates, as one would expect, but most of the ATTs remain statistically significant, which is reassuring.

Another concern may be related to the fact that we express most of the outcome variables in logarithmic form, even though several include zero or–in the case of crop income–even negative observations (see S3 Table), for which the logarithm is not defined. It should be noted that the number of households with zero or negative observations for the outcome variables is small and mostly confined to the group of non-adopters. Very few of these households with zero or negative observations were selected as relevant matches based on their propensity scores. Nevertheless, we reran the PSM estimates also in linear form, without taking logs, and obtained similar results. For some of the outcome variables, the estimated ATTs even increased (see S8 Table in the supplementary information). With the outcome variables expressed in linear form, the digital extension effect on crop productivity increased to 21%, and the effect on crop income increased to around 40%. Hence, using the log-transformation seems to lead to conservative estimates.

We cautiously conclude that digital technologies that use data from farms to provide personalized information are effective in terms of helping farmers to make better cropping, technology, and input decisions. The information provided by the digital extension services on the best types of crops to grow, the appropriate methods of cultivation, the optimal timing of the different operations, and the suitable input regimes seems to help farmers allocate their resources more efficiently. This leads to the use of better technologies and higher input intensities, thus increasing cop productivity and income.

### 4.5 Sensitivity to potential hidden bias

The PSM estimates of the ATTs may still be biased if any unobserved variables affect treatment assignment and are also correlated with the outcome variables. Using the PSM estimates, we analyse how sensitive our results are to such potential hidden bias by calculating Rosenbaum bounds [66]. Rosenbaum bounds estimate critical levels of hidden bias (Γ) for the ATTs at which the conclusion of a positive treatment effect would have to be challenged. The results are shown in Table 6.

**Table 6:** Critical level of hidden bias (Γ).

	Nearest neighbour matching	Radius matching	Kernel matching
Number of crops grown	1.30	1.15	1.20
Seed expenditure per acre (log)	1.15	1.45	1.40
Fertilizer expenditure per acre (log)	1.30	1.40	1.45
Pesticide expenditure per acre (log)	1.30	1.45	1.45
Total input expenditure per acre (log)	1.35	1.60	1.65
Crop productivity (log)	1.40	1.50	1.50
Crop commercialization	1.40	1.30	1.30
Crop income (log)	1.30	1.45	1.50

The robustness of the ATTs to potential hidden bias varies across the outcome variables and the three matching methods (Table 6). A value of 1.30 for the number of crops grown and the nearest neighbour matching algorithm means that the respective ATT would remain positive and significant at the 90% level even if there were hidden bias up to a magnitude of 30% (meaning 30% systematic difference between treatment and control group in terms of unobserved factors even after matching). Only if there were hidden biases of more than 30%, the ATT would turn insignificant. Remember that when estimating propensity scores for matching we did not only include many farm and household covariates but also WTP as a proxy for relevant unobserved factors. Against this background, we expect that any remaining hidden bias would be lower than 30%. For many of the other outcome variables, the critical levels for hidden bias are larger than 1.30 (Table 6), meaning that the conclusion of significantly positive associations is robust.

### 4.6 Additional robustness checks

To further test the robustness of the PSM estimates, we used several alternative methods, as explained in section 3.3. The ATTs obtained with the IPWRA method are shown in S9 Table in the supplementary information. They are very similar to those obtained with PSM in terms of both their size and significance levels. The OLS results with the inclusion of WTP to control for unobserved heterogeneity are shown in S10 Table. These results are also very similar to both the PSM and IPWRA results.

Further, to show that our results are not driven by omitted variable bias we estimate Oster bounds using multiple specifications. To calculate the bounds to the true value of the treatment effect, we need to fix two parameters, namely (i) δ, which indicates the relative importance of unobservables compared to observables, and (ii) R_max_, which is the maximum R-squared that can be achieved from a hypothetical regression that controls for all variables including unobserved factors. Oster [59] argues that $\tilde{R}<R_{max}<1$, where $\tilde{R}$ is the R-squared from the estimated equation. The standard case in the literature is to use R_max_ equal to 1.3 times the R-square value of the estimated equation with full controls [59,67–69]. We follow this convention and present results with varying assumptions for δ in S10 Table. In all cases, the treatment bounds do not include the value zero, except for crop income when large values for δ are assumed. Overall, these results are reassuring.

Finally, we present results of coarsened exact matching (CEM) in S12 and S13 Tables in the supplementary information. Here, we use a smaller set of covariates for matching, as the inclusion of many variables leads to fewer exact matches. Instead, we control for remaining imbalances after matching using linear regression models. For CEM, we coarsen continuous variables such as age, household size, and land size, into sub-categories. Age is coarsened into bins of (20–40), (40–60), and (above 60 years) to categorize household heads as young adult, middle aged, and older heads respectively. The binning strategy for operated land is small farms (2.5–5 acres), medium farms (5–10 acres), and large farms (>10 acres), and the binning strategy for household size is 1(0–4 household members) and 0 (more than 4 members). The remaining variables were already in dichotomous form, so we use those variables as in the original data. Each household is assigned a binning signature based on the coarsened and dichotomous variables and then exactly matched with households in the other group. The ATT estimation results are shown in S13 Table. They are very similar to the PSM results discussed above.

Based on the various robustness checks, we conclude that our findings are quite robust to variations in matching approaches, estimation methods, and possible unobserved heterogeneity.

### 4.7 Limitations

While we have tried hard to control and test for potential biases, several limitations need to be pointed out. First, our analysis relies on cross-section observational data where the establishment of causality is difficult. Hence, our results should be interpreted as associations between digital extension adoption and agricultural performance and not as fully identified net effects. Although we addressed issues of endogeneity to the extent possible, follow-up research with panel data and/or experimental approaches could be useful to further improve the identification strategy. Second, the results from one example of an agri-tech platform in one farmer organization in eastern India should not be generalized. Additional studies in other contexts would be useful to increase the external validity of the results. Third, we concentrated on a few outcome variables related to crop production and crop income, as this is what the agri-tech platform in the study region focuses on. Crop productivity and crop income are not comprehensive measures of household welfare. Future studies could analyse other important outcomes related to total household income, food security, time allocation, and gender roles, among others. Fourth, we looked at effectiveness in terms of improving agricultural performance without considering the costs of providing and using digital services. Studies on cost-effectiveness would be useful to gain further policy-relevant insights.

## 5. Conclusion

Traditionally, the diffusion of agricultural information in developing countries has been promoted through the public extension service, wherein extension agents visit and educate individual farmers or farmer groups. This traditional way of information dissemination has two major drawbacks. First, as personal visits are associated with high transaction costs, only a very limited number of farmers can be reached. Second, the information provided through this channel is often generic and not necessarily well adapted to farmers’ specific needs and conditions. Using digital approaches and technologies can potentially improve the effectiveness of agricultural extension services by reducing transaction costs and improving the quality of the information provided. Data-driven algorithms can be used to tailor information to the specific conditions of individual farmers. However, research on the actual effectiveness of such personalized digital extension approaches in the small farm sector is still very limited.

In this study, we collected and used data from smallholder vegetable farmers in eastern India to analyse the association between using personalized digital extension services and agricultural performance. The digital agri-tech platform that had recently been launched in the study region provides advice on which types of crops to grow and inputs to use, considering the weather, soil, and other agronomic as well as socioeconomic conditions of each farmer. For the analysis, we looked at various outcome variables such as crop diversity, input intensity, crop productivity, levels of commercialization, and crop income.

As farmers decided themselves whether to adopt the digital extension services, the evaluation needed to deal with possible issues of selection bias. We employed propensity score matching to reduce bias due to observed heterogeneity between digital extension service adopters and non-adopters. Moreover, we used a willingness to pay (WTP) variable as an additional covariate in the propensity score model to also reduce possible bias from unobserved heterogeneity. As robustness checks, we used and compared different matching methods (PSM and CEM), employed other econometric tools (OLS and IPWRA), and corrected for multiple hypothesis testing.

Our results show that adopting personalized digital extension services is positively associated with several or the agricultural performance indicators. After controlling for confounding factors, digital extension adopters were found to have 15–20% higher input intensity, 18% higher crop productivity, and 18–29% higher crop incomes than non-adopters. These findings suggest that digital approaches and technologies can be effective tools to improve personalized agricultural extension and promote smallholder productivity and income.

The associations identified here are relatively large in magnitude and larger than what several other studies on digital agricultural extension found [20,21,34]. Given that we cannot completely rule out any remaining bias, our associations may potentially be overestimated. On the other hand, as discussed above, most existing studies evaluated digital extension services providing generic advice, not personalized advice as analysed here. Personalized advice that is tailored to farmers’ specific needs and conditions should be expected to have larger effects. The only existing studies evaluating the effects of personalized digital extension are Arouna et al. [38] and Oyinbo et al. [39]. Arouna et al. [38] found that personalized fertilizer recommendations provided through digital tools lead to yield increases of 7% and profit increases of 10% in Nigeria. Our estimates are larger, but the Farmex platform analyzed here is more comprehensive and provides digital advice not only on fertilizer use but on several other aspects as well, including crop choices and pest control, among others. Hence, larger estimates are not unexpected, even though we stress that the exact magnitude of the associations found here should be interpreted with some caution. In any case, our results suggest that personalized digital extension services have promising potential to promote agricultural development in the small farm sector.

The agri-tech platform in the study region in India is still relatively simple and can be further improved. So far, the services provided concentrate only on the input side, although there are plans to extend the services and use the platform to also link farmers directly to retailers and consumers. Technological developments in areas such as open-source software and big data analytics may further improve the types of services provided. In the Indian example analysed here, the services are provided free of charge to farmers through a social business enterprise. However, if measurable benefits are confirmed, farmers may also be willing to pay a certain amount for such digital services, as indicated by significantly positive WTP estimates in our analysis.

Nevertheless, some public support may be needed to make digital extension services effective and accessible for a large number of farmers. For instance, certain infrastructure elements–such as roads, electricity, telephone network, and internet coverage–are important preconditions for digital service providers to become active in a region. In addition, a minimum level of computer and digital literacy is required either among farmers or at least among local intermediaries. Therefore, from a policy perspective, investments in rural road and ICT infrastructure, in promoting digital literacy among rural households, and in creating an enabling business environment for related entrepreneurial activities are important steps towards fostering agricultural innovation and equitable growth in the small-farm sector.

## Supporting information

S1 Data (XLS)

S1 Dataset Dataset dictionary. (DOCX)

S1 Table Number of farmers by village and sample size. (DOCX)

S2 Table Summary statistics of control group farmers by FPO membership. (DOCX)

S3 Table Summary statistics of full sample. (DOCX)

S4 Table Correction for multiple inferences of the treatment effects. (DOCX)

S5 Table PSM estimates with bootstrapped standard errors with 10,000 replications (robustness check). (DOCX)

S6 Table PSM estimates excluding potentially endogenous variables (robustness check). (DOCX)

S7 Table PSM estimates excluding WTP and other potentially endogenous variables (robustness check). (DOCX)

S8 Table PSM estimates with outcome variables in linear form. (DOCX)

S9 Table Association between adopting digital extension services and agricultural performance (IPWRA results). (DOCX)

S10 Table OLS estimates with WTP as additional control variable (robustness check). (DOCX)

S11 Table Oster bounds. (DOCX)

S12 Table L1 measure of imbalance before and after coarsened exact matching. (DOCX)

S13 Table Estimated associations based on coarsened exact matched data. (DOCX)
