# Supplementary material for: Personalized digital extension services and agricultural performance: Evidence from smallholder farmers in India
Source: PLoS One. 2021 Oct 28;16(10):e0259319. doi: 10.1371/journal.pone.0259319 (PMC8553076; doi:10.1371/journal.pone.0259319)
Supplement: S1 Dataset — (DOCX) [file pone.0259319.s002.docx]

**Dataset dictionary**

| **Variable Name** | **Definition** |
| --- | --- |
| HHID | Household ID |
| BLOCK | Block Name |
| VILLAGE | Village Code |
| GENERAL | General caste (1=Yes) |
| OBC | Other backward caste (1=Yes) |
| SC | Scheduled caste (1=Yes) |
| ST | Scheduled tribe (1=Yes) |
| FPO | FPO Member |
| HH_Sex | Male household head (1=Yes) |
| HH_AGE | Age of household head (years) |
| PRIMARY | Primary school: highest education of adult male (1=Yes) |
| SECONDARY | Secondary school: highest education of adult male (1=Yes) |
| BA_MA | Bachelor or Masters: highest education of adult male (1=Yes) |
| ILLITERATE | Illiterate: highest education of adult male (1=Yes) |
| NANIMALS | Livestock ownership (livestock units) |
| OPLAND | Operated land (acres) |
| IRR | Irrigation ratio (%) |
| OFF_FARM | Off farm income (1=Yes) |
| HH_Mobile | Household head owns a mobile phone (1=Yes) |
| NPERSON | Household size (number) |
| FARMEX | Household uses digital extensions services (1=Yes) |
| WTP | Willingness to pay for digital agri-tech platform services |
| NCROP | Number of crops grown |
| AGRI_INCOME | Crop income (Rs/Acre) |
| YIELD | Yield (Rs/Acre) |
| PEER_CASTE | Peer group |
| SEED_INTENSITY | Seed expenditure per acre (Rs/Acre) |
| FERT_INTENSITY | Fertilizer expenditure per acre (Rs/Acre) |
| PEST_INTENSITY | Pesticides expenditure per acre (Rs/Acre) |
| INPUT_INTENSITY | Total input expenditure per acre (Rs/Acre) |
| AGRI_COMM | Commercialization (share of farm output sold 0-1) |
| DISTMARKET | Average distance to input and output market (km) |
