# Supplementary material for: Personalized digital extension services and agricultural performance: Evidence from smallholder farmers in India
Source: PLoS One. 2021 Oct 28;16(10):e0259319. doi: 10.1371/journal.pone.0259319 (PMC8553076; doi:10.1371/journal.pone.0259319)
Supplement: S1 Table — (DOCX) [file pone.0259319.s003.docx]

**Table S1: Number of farmers by village and sample size**

|  |  | Population | | | Sample | |
| --- | --- | --- | --- | --- | --- | --- |
| Block | **Village** | **FPO vegetable farmers** | **Non-FPO**  **vegetable farmers** | **Non-FPO non-vegetable farmers** | **FPO vegetable farmers** | **Non-FPO**  **vegetable farmers** |
| Betnoti | Rangada | 43  (0.10) | 54  (0.05) | 24 | 30 | 10 |
|  | Sribatsapur | 52  (0.13) | 106  (0.10) | 63 | 37 | 19 |
|  | Demphauda | 35  (0.08) | 101  (0.10) | 57 | 23 | 19 |
|  | Madhunanda | 52  (0.13) | 61  (0.06) | 10 | 36 | 11 |
|  | Khadikapada | 16  (0.04) | 92  (0.09) | 144 | 11 | 16 |
|  | Nakhara | 63  (0.15) | 114  (0.11) | 4 | 45 | 20 |
|  | Khandadeulia | 50  (0.12) | 148  (0.14) | 12 | 36 | 27 |
|  | Panchaputuli | 22  (0.05) | 20  (0.02) | 6 | 15 | 4 |
|  | Raikama | 48  (0.12) | 193  (0.19) | 100 | 34 | 35 |
|  | Badakhirapada | 31  (0.08) | 151  (0.15) | 8 | 21 | 27 |
| Total Betnoti |  | **412** | **1040** | **428** | **288** | **188** |
| Badasahi | Bhanjabati | 31  (0.07) | 63  (0.04) | 62 | 21 | 11 |
|  | Haripur | 65  (0.14) | 491  (0.28) | 255 | 45 | 87 |
|  | Kuliana | 36  (0.08) | 121  (0.07) | 43 | 25 | 21 |
|  | Baghuapal | 40  (0.09) | 61  (0.03) | 8 | 28 | 11 |
|  | Singtia | 23  (0.05) | 313  (0.18) | 150 | 16 | 55 |
|  | Mankadapal | 30  (0.07) | 3  (0.002) | 8 | 21 | 1 |
|  | Sakua | 64  (0.14) | 142  (0.08) | 109 | 44 | 25 |
|  | Chakradharpur | 28  (0.07) | 157  (0.099 | 239 | 19 | 27 |
|  | Khuntapal | 55  (0.12) | 211  (0.12) | 40 | 38 | 36 |
|  | Sorisakatha | 84  (0.18) | 221  (0.12) | 9 | 58 | 40 |
| Total Badasahi |  | **456** | **1783** | **923** | **315** | **314** |

Note: Numbers in parentheses are the proportions relative to the total households in each block.
