# Supplementary material for: Personalized digital extension services and agricultural performance: Evidence from smallholder farmers in India
Source: PLoS One. 2021 Oct 28;16(10):e0259319. doi: 10.1371/journal.pone.0259319 (PMC8553076; doi:10.1371/journal.pone.0259319)
Supplement: S2 Table — (DOCX) [file pone.0259319.s004.docx]

**Table S2: Summary statistics of control group farmers by FPO membership**

|  | **FPO members** | | **Non-members** | |
| --- | --- | --- | --- | --- |
|  | **Mean** | **SD** | **Mean** | **SD** |
| Age of household head (years) | 52.72 | 11.69 | 49.03 | 15.12 |
| Male household head (dummy) | 0.92 | 0.27 | 0.90 | 0.30 |
| Household head owns a mobile phone (dummy) | 0.67 | 0.47 | 0.69 | 0.46 |
| Illiterate (dummy) | 0.09 | 0.29 | 0.10 | 0.29 |
| Primary school (dummy) | 0.25 | 0.44 | 0.28 | 0.45 |
| Secondary school (dummy) | 0.43 | 0.50 | 0.39 | 0.49 |
| Bachelor or Masters (dummy) | 0.21 | 0.41 | 0.17 | 0.38 |
| Scheduled tribe (dummy) | 0.18 | 0.39 | 0.19 | 0.39 |
| Scheduled caste (dummy) | 0.16 | 0.37 | 0.22 | 0.41 |
| Other backward classes (dummy) | 0.46 | 0.50 | 0.43 | 0.50 |
| General caste (dummy) | 0.20 | 0.40 | 0.17 | 0.38 |
| Household size (number) | 3.76 | 1.43 | 3.55 | 1.44 |
| Operated land (acres) | 5.09 | 3.78 | 3.78 | 3.90 |
| Irrigation ratio (%) | 51.36 | 34.31 | 47.22 | 39.87 |
| Observations | 138 |  | 502 |  |
