# Supplementary material for: Personalized digital extension services and agricultural performance: Evidence from smallholder farmers in India
Source: PLoS One. 2021 Oct 28;16(10):e0259319. doi: 10.1371/journal.pone.0259319 (PMC8553076; doi:10.1371/journal.pone.0259319)
Supplement: S3 Table — (DOCX) [file pone.0259319.s005.docx]

**Table S3: Summary statistics of full sample**

|  | **Mean** | **SD** | **Min** | **Max** |
| --- | --- | --- | --- | --- |
| Age of household head (years) | 50.50 | 13.40 | 21.00 | 96.00 |
| Male household head (dummy) | 0.94 | 0.24 | 0.00 | 1.00 |
| Household head owns a mobile phone (dummy) | 0.73 | 0.44 | 0.00 | 1.00 |
| Illiterate ^a^ (dummy) | 0.08 | 0.27 | 0.00 | 1.00 |
| Primary school ^a^ (dummy) | 0.25 | 0.43 | 0.00 | 1.00 |
| Secondary school ^a^ (dummy) | 0.42 | 0.49 | 0.00 | 1.00 |
| Bachelor or Masters ^a^ (dummy) | 0.23 | 0.42 | 0.00 | 1.00 |
| Scheduled tribe (dummy) | 0.15 | 0.35 | 0.00 | 1.00 |
| Scheduled caste (dummy) | 0.17 | 0.38 | 0.00 | 1.00 |
| Other backward classes (dummy) | 0.49 | 0.50 | 0.00 | 1.00 |
| General caste (dummy) | 0.19 | 0.39 | 0.00 | 1.00 |
| Household size (number) | 3.75 | 1.43 | 1.00 | 11.00 |
| Land ownership (acres) | 1.33 | 1.42 | 0.00 | 12.00 |
| Operated land (acres) | 4.78 | 4.03 | 0.03 | 36.00 |
| Operational land < 2.5 acres (dummy) | 0.32 | 0.47 | 0.00 | 1.00 |
| Operational land 2.5-5 acres (dummy) | 0.30 | 0.46 | 0.00 | 1.00 |
| Operational land 5-10 acres (dummy) | 0.29 | 0.45 | 0.00 | 1.00 |
| Operational land > 10 acres (dummy) | 0.09 | 0.29 | 0.00 | 1.00 |
| Irrigation ratio (%) | 50.84 | 37.47 | 0.00 | 116.75 |
| FPO member (dummy) | 0.55 | 0.50 | 0.00 | 1.00 |
| Subscriber to digital advisory service (dummy) | 0.43 | 0.50 | 0.00 | 1.00 |
| Livestock ownership (livestock units) | 1.23 | 1.18 | 0.00 | 21.50 |
| Average distance to input and output market (km) | 5.03 | 4.10 | 0.00 | 25.33 |
| Willingness to pay for digital agri-tech platform services | 219.84 | 397.09 | 10.00 | 5000.00 |
| Peer group ^b^ | 12.59 | 9.29 | 0.00 | 32.00 |
| Off farm income (dummy) | 0.65 | 0.48 | 0.00 | 1.00 |
| **Outcome variables** |  |  |  |  |
| Number of crops grown | 7.25 | 4.71 | 1.00 | 34.00 |
| Seed expenditure (1,000 Rupees/acre) | 0.75 | 0.94 | 0.00 | 7.462 |
| Fertilizer expenditure (1,000 Rupees/acre) | 1.76 | 1.51 | 0.00 | 13.72 |
| Pesticides expenditure (1,000 Rupees/acre) | 0.67 | 0.86 | 0.00 | 10.45 |
| Input expenditure (1,000 Rupees/acre) | 3.18 | 2.91 | 0.00 | 31.34 |
| Crop productivity (1,000 Rupees/acre) | 15.10 | 16.28 | 0.00 | 221.60 |
| Commercialization (share of farm output sold 0-1) | 0.44 | 0.32 | 0.00 | 1.89 |
| Crop income (1,000 Rupees) | 35.58 | 66.52 | -116.25 | 625.51 |
| Observations | 1028 |  |  |  |

^a^ Highest education level of adult male. ^b^ Number of households within the village from the same caste who adopted digital extension services
