# Supplementary material for: Personalized digital extension services and agricultural performance: Evidence from smallholder farmers in India
Source: PLoS One. 2021 Oct 28;16(10):e0259319. doi: 10.1371/journal.pone.0259319 (PMC8553076; doi:10.1371/journal.pone.0259319)
Supplement: S4 Table — (DOCX) [file pone.0259319.s006.docx]

**Table S4: Correction for multiple inferences of the treatment effects**

|  | **(1)** | **(2)** | **(3)** | **(4)** | **(5)** |
| --- | --- | --- | --- | --- | --- |
|  | **Nearest neighbour matching** | **Radius matching** | **Kernel based matching** | **IPWR** | **OLS** |
| *Panel A: Production diversity* |  |  |  |  |  |
| Unadjusted p-value | 0.006 | 0.007 | 0.002 | 0.036 | 0.002 |
| Bonferroni adjusted p-value | 0.048 | 0.056 | 0.016 | 0.288 | 0.016 |
| Holm adjusted p-value | 0.048 | 0.042 | 0.016 | 0.042 | 0.010 |
| *Panel B: Seed expenditure (log)* | |  |  |  |  |
| Unadjusted p-value | 0.153 | 0.040 | 0.032 | 0.000 | 0.005 |
| Bonferroni adjusted p-value | 0.999 | 0.320 | 0.256 | 0.000 | 0.040 |
| Holm adjusted p-value | 0.153 | 0.096 | 0.080 | 0.000 | 0.015 |
| Sharpened q-value | 0.153 | 0.046 | 0.035 | 0.001 | 0.007 |
| *Panel C: Fertilizer expenditure (log)* | |  |  |  |  |
| Unadjusted p-value | 0.028 | 0.018 | 0.020 | 0.021 | 0.001 |
| Bonferroni adjusted p-value | 0.224 | 0.144 | 0.160 | 0.168 | 0.008 |
| Holm adjusted p-value | 0.112 | 0.090 | 0.080 | 0.042 | 0.007 |
| Sharpened q-value | 0.039 | 0.032 | 0.028 | 0.007 | 0.003 |
| *Panel D: Pesticide expenditure (log)* | |  |  |  |  |
| Unadjusted p-value | 0.041 | 0.024 | 0.021 | 0.005 | 0.003 |
| Bonferroni adjusted p-value | 0.328 | 0.192 | 0.168 | 0.040 | 0.024 |
| Holm adjusted p-value | 0.112 | 0.096 | 0.080 | 0.020 | 0.012 |
| Sharpened q-value | 0.047 | 0.032 | 0.028 | 0.004 | 0.005 |
| *Panel E: Total input expenditure (log)* | |  |  |  |  |
| Unadjusted p-value | 0.014 | 0.003 | 0.002 | 0.001 | 0.000 |
| Bonferroni adjusted p-value | 0.112 | 0.024 | 0.016 | 0.008 | 0.000 |
| Holm adjusted p-value | 0.070 | 0.024 | 0.016 | 0.007 | 0.000 |
| Sharpened q-value | 0.028 | 0.012 | 0.006 | 0.002 | 0.001 |
| *Panel F: Crop yield (log)* |  |  |  |  |  |
| Unadjusted p-value | 0.006 | 0.003 | 0.002 | 0.001 | 0.001 |
| Bonferroni adjusted p-value | 0.048 | 0.024 | 0.016 | 0.008 | 0.008 |
| Holm adjusted p-value | 0.048 | 0.024 | 0.016 | 0.007 | 0.007 |
| Sharpened q-value | 0.024 | 0.032 | 0.006 | 0.002 | 0.003 |
| *Panel G: Crop commercialization* | |  |  |  |  |
| Unadjusted p-value | 0.009 | 0.046 | 0.035 | 0.005 | 0.007 |
| Bonferroni adjusted p-value | 0.072 | 0.368 | 0.280 | 0.040 | 0.056 |
| Holm adjusted p-value | 0.054 | 0.096 | 0.080 | 0.020 | 0.015 |
| Sharpened q-value | 0.024 | 0.046 | 0.035 | 0.004 | 0.008 |
| *Panel H: Crop income* |  |  |  |  |  |
| Unadjusted p-value | 0.029 | 0.024 | 0.012 | 0.001 | 0.028 |
| Bonferroni adjusted p-value | 0.232 | 0.192 | 0.096 | 0.008 | 0.224 |
| Holm adjusted p-value | 0.112 | 0.096 | 0.060 | 0.007 | 0.028 |
| Sharpened q-value | 0.039 | 0.046 | 0.024 | 0.002 | 0.028 |

Note: Columns (1) to (3) present unadjusted and adjusted *p*-values (*q*-values) for the PSM results in Table 5. Columns (4) and (5) present unadjusted and adjusted *p*-values (*q*-values) for results shown in Tables S9 and S10.
