# Supplementary material for: Personalized digital extension services and agricultural performance: Evidence from smallholder farmers in India
Source: PLoS One. 2021 Oct 28;16(10):e0259319. doi: 10.1371/journal.pone.0259319 (PMC8553076; doi:10.1371/journal.pone.0259319)
Supplement: S5 Table — (DOCX) [file pone.0259319.s007.docx]

**Table S5: PSM estimates with bootstrapped standard errors with 10,000 replications (robustness check)**

|  | **Nearest neighbour matching** | | **Radius**  **matching** | | **Kernel**  **matching** | |
| --- | --- | --- | --- | --- | --- | --- |
| **Outcome variable** | **ATT** | **SE** | **ATT** | **SE** | **ATT** | **SE** |
| Number of crops grown | 1.211*** | (0.434) | 1.012*** | (0.383) | 1.095*** | (0.358) |
| Seed expenditure per acre (log) | 0.170 | (0.117) | 0.212** | (0.098) | 0.198** | (0.094) |
| Fertilizer expenditure per acre (log) | 0.161** | (0.077) | 0.153** | (0.062) | 0.153** | (0.062) |
| Pesticide expenditure per acre (log) | 0.199** | (0.099) | 0.204** | (0.085) | 0.198** | (0.084) |
| Total expenditure per acre (log) | 0.188** | (0.078) | 0.198*** | (0.066) | 0.197*** | (0.065) |
| Crop productivity (log) | 0.175*** | (0.066) | 0.176*** | (0.057) | 0.177*** | (0.057) |
| Crop commercialization | 0.074*** | (0.029) | 0.048** | (0.024) | 0.049** | (0.024) |
| Crop income (log) | 0.285** | (0.130) | 0.243** | (0.107) | 0.265** | (0.105) |

ATT: average treatment effect on the treated. PSM: propensity score matching. Bootstrapped standard errors with 10,000 replications are shown in parentheses. * Significant at 10% level, ** Significant at 5% level, ***Significant at 1% level
