# Supplementary material for: Personalized digital extension services and agricultural performance: Evidence from smallholder farmers in India
Source: PLoS One. 2021 Oct 28;16(10):e0259319. doi: 10.1371/journal.pone.0259319 (PMC8553076; doi:10.1371/journal.pone.0259319)
Supplement: S6 Table — (DOCX) [file pone.0259319.s008.docx]

**Table S6: PSM estimates excluding potentially endogenous variables (robustness check)**

|  | **Nearest neighbour matching** | | **Radius**  **matching** | | **Kernel**  **matching** | |
| --- | --- | --- | --- | --- | --- | --- |
| **Outcome variable** | **ATT** | **SE** | **ATT** | **SE** | **ATT** | **SE** |
| Number of crops grown | 1.061** | (0.422) | 1.072*** | (0.345) | 1.120*** | (0.350) |
| Seed expenditure per acre (log) | 0.253** | (0.116) | 0.240** | (0.098) | 0.230** | (0.092) |
| Fertilizer expenditure per acre (log) | 0.164** | (0.079) | 0.168*** | (0.061) | 0.161** | (0.063) |
| Pesticide expenditure per acre (log) | 0.239** | (0.098) | 0.235*** | (0.081) | 0.223*** | (0.082) |
| Total expenditure per acre (log) | 0.221*** | (0.076) | 0.219** | (0.064) | 0.211*** | (0.064) |
| Crop productivity (log) | 0.185*** | (0.071) | 0.189*** | (0.058) | 0.182*** | (0.058) |
| Crop commercialization | 0.050* | (0.027) | 0.049** | (0.023) | 0.051** | (0.023) |
| Crop income (log) | 0.244* | (0.126) | 0.261** | (0.102) | 0.273** | (0.109) |

Note: Ownership of mobile phones, off-farm income, and peer group were the three potentially endogenous variables excluded in these calculations. ATT: Average treatment effect on the treated. Bootstrapped standard errors with 1000 replications are shown in parentheses. * Significant at 10% level, ** Significant at 5% level, ***Significant at 1% level
