# Supplementary material for: Personalized digital extension services and agricultural performance: Evidence from smallholder farmers in India
Source: PLoS One. 2021 Oct 28;16(10):e0259319. doi: 10.1371/journal.pone.0259319 (PMC8553076; doi:10.1371/journal.pone.0259319)
Supplement: S7 Table — (DOCX) [file pone.0259319.s009.docx]

**Table S7: PSM estimates excluding WTP and other potentially endogenous variables (robustness check)**

|  | **Nearest neighbour matching** | | **Radius matching** | | **Kernel matching** | |
| --- | --- | --- | --- | --- | --- | --- |
| **Outcome variable** | **ATT** | **SE** | **ATT** | **SE** | **ATT** | **SE** |
| Number of crops grown | 1.156*** | (0.411) | 1.053*** | (0.382) | 1.090*** | (0.352) |
| Seed expenditure per acre (log) | 0.173 | (0.114) | 0.153 | (0.094) | 0.160* | (0.089) |
| Fertilizer expenditure per acre (log) | 0.073 | (0.078) | 0.115* | (0.061) | 0.114* | (0.060) |
| Pesticide expenditure per acre (log) | 0.173 | (0.108) | 0.153* | (0.083) | 0.136 | (0.084) |
| Total expenditure per acre (log) | 0.124 | (0.086) | 0.152** | (0.064) | 0.150** | (0.063) |
| Crop productivity (log) | 0.151** | (0.068) | 0.178*** | (0.059) | 0.177*** | (0.057) |
| Crop commercialization | 0.048* | (0.028) | 0.049** | (0.022) | 0.055** | (0.022) |
| Crop income (log) | 0.302** | (0.129) | 0.309*** | (0.105) | 0.313*** | (0.101) |

Note: WTP for digital extension services, ownership of mobile phones, off-farm income, and peer group were excluded in these calculations. ATT: Average treatment effect on the treated. Bootstrapped standard errors with 1000 replications are shown in parentheses. * Significant at 10% level, ** Significant at 5% level, ***Significant at 1% level
