# Supplementary material for: Personalized digital extension services and agricultural performance: Evidence from smallholder farmers in India
Source: PLoS One. 2021 Oct 28;16(10):e0259319. doi: 10.1371/journal.pone.0259319 (PMC8553076; doi:10.1371/journal.pone.0259319)
Supplement: S8 Table — (DOCX) [file pone.0259319.s010.docx]

**Table S8**: **PSM estimates with outcome variables in linear form**

|  | **Nearest neighbour matching** | | **Radius matching** | | **Kernel matching** | |
| --- | --- | --- | --- | --- | --- | --- |
| **Outcome variable** | **ATT** | **SE** | **ATT** | **SE** | **ATT** | **SE** |
| Number of crops grown | 1.211*** | (0.425) | 1.011*** | (0.387) | 1.095*** | (0.340) |
| Seed expenditure (Rupees/acre) | 36.028 | (91.105) | 67.238 | (66.604) | 67.820 | (63.992) |
| Fertilizer expenditure (Rupees/acre) | 171.598 | (126.407) | 149.823 | (106.567) | 138.703 | (103.333) |
| Pesticide expenditure (Rupees/acre) | 49.042 | (68.048) | 52.329 | (56.855) | 44.888 | (60.177) |
| Total expenditure (Rupees/acre) | 256.668 | (240.106) | 269.389 | (200.414) | 251.411 | (190.066) |
| Crop productivity (Rupees/acre) | 2802.004** | (1231.727) | 2821.488** | (1111.778) | 2720.797** | (1122.908) |
| Crop commercialization | 0.074*** | (0.028) | 0.048** | (0.024) | 0.049** | (0.024) |
| Crop income (Rupees) | 14531.933** | (5814.817) | 13374.854*** | (5058.407) | 13762.057*** | (5141.309) |

ATT: average treatment effect on the treated. PSM: propensity score matching. Bootstrapped standard errors with 1,000 replications are shown in parentheses. * Significant at 10% level, ** Significant at 5% level, ***Significant at 1% level
