# Supplementary material for: Personalized digital extension services and agricultural performance: Evidence from smallholder farmers in India
Source: PLoS One. 2021 Oct 28;16(10):e0259319. doi: 10.1371/journal.pone.0259319 (PMC8553076; doi:10.1371/journal.pone.0259319)
Supplement: S9 Table — (DOCX) [file pone.0259319.s011.docx]

**Table S9: Association between adopting digital extension services and agricultural performance (IPWRA results)**

| **Outcome variable** | **ATT** | **Robust SE** |
| --- | --- | --- |
| Number of crops grown | 0.793** | (0.378) |
|  | [0.010] |  |
| Seed expenditure per acre (log) | 0.256*** | (0.073) |
|  | [0.001] |  |
| Fertilizer expenditure per acre (log) | 0.121** | (0.052) |
|  | [0.007] |  |
| Pesticide expenditure per acre (log) | 0.182*** | (0.065) |
|  | [0.004] |  |
| Total expenditure per acre (log) | 0.164*** | (0.052) |
|  | [0.002] |  |
| Crop productivity (log) | 0.178*** | (0.053) |
|  | [0.002] |  |
| Crop commercialization | 0.060*** | (0.022) |
|  | [0.004] |  |
| Crop income (log) | 0.291*** | (0.090) |
|  | [0.002] |  |

ATT: average treatment effect on the treated. IPWRA: inverse-probability weighted regression adjustment.

* Significant at 10% level, ** Significant at 5% level, ***Significant at 1% level. Multiple hypotheses corrected sharpened *q*-values are presented in square brackets. Unadjusted *p*-values and Bonferroni and Holm adjusted *p*-values are shown in Table S4.
