# Supplementary material for: Personalized digital extension services and agricultural performance: Evidence from smallholder farmers in India
Source: PLoS One. 2021 Oct 28;16(10):e0259319. doi: 10.1371/journal.pone.0259319 (PMC8553076; doi:10.1371/journal.pone.0259319)
Supplement: S10 Table — (DOCX) [file pone.0259319.s012.docx]

**Table S10: OLS estimates with WTP as additional control variable (robustness check)**

|  | **(1)** | **(2)** | **(3)** | **(4)** | **(5)** | **(6)** | **(7)** | **(8)** |
| --- | --- | --- | --- | --- | --- | --- | --- | --- |
|  | **Number of crops** | **Seed expenditure (log)** | **Fertilizer expenditure (log)** | **Pesticide expenditure (log)** | **Input expenditure (log)** | **Crop productivity (log)** | **Crop commercialization (0-1)** | **Crop income (log)** |
| Digital extension (dummy) | 0.934*** | 0.206*** | 0.164*** | 0.190*** | 0.189*** | 0.170*** | 0.052*** | 0.200** |
|  | (0.295) | (0.073) | (0.050) | (0.064) | (0.051) | (0.053) | (0.019) | (0.091) |
|  | [0.004] | [0.007] | [0.003] | [0.005] | [0.001] | [0.002] | [0.008] | [0.028] |
| WTP and other controls included ^a^ | Yes | Yes | Yes | Yes | Yes | Yes | Yes | Yes |
| Village fixed effects included | Yes | Yes | Yes | Yes | Yes | Yes | Yes | Yes |
| Observations | 1028 | 933 | 1005 | 938 | 1018 | 1024 | 1024 | 860 |
| R-squared | 0.295 | 0.354 | 0.322 | 0.389 | 0.363 | 0.202 | 0.305 | 0.395 |

* Significant at 10% level, ** Significant at 5% level, ***Significant at 1% level. Robust standard errors are shown in parentheses. Multiple hypotheses corrected sharpened *q*-values are shown in square brackets. ^a^ Controls included are the same as in Table 3.
