# Supplementary material for: Personalized digital extension services and agricultural performance: Evidence from smallholder farmers in India
Source: PLoS One. 2021 Oct 28;16(10):e0259319. doi: 10.1371/journal.pone.0259319 (PMC8553076; doi:10.1371/journal.pone.0259319)
Supplement: S11 Table — (DOCX) [file pone.0259319.s013.docx]

**Table S11: Oster bounds**

|  | **(1)** | **(2)** | **(3)** | **(4)** | **(5)** |
| --- | --- | --- | --- | --- | --- |
| **Outcome variable** | **Baseline coefficient** |  | **Bias-adjusted coefficient** | | |
|  |  | (δ=0.5) | (δ=1) | (δ=1.2) | (δ=1.5) |
| Number of crops grown | 0.934*** | 0.678 | 0.401 | 0.282 | 0.087 |
| Seed expenditure per acre (log) | 0.206*** | 0.211 | 0.216 | 0.219 | 0.222 |
| Fertilizer expenditure per acre (log) | 0.164*** | 0.176 | 0.189 | 0.195 | 0.204 |
| Pesticide expenditure per acre (log) | 0.190*** | 0.203 | 0.216 | 0.222 | 0.231 |
| Total expenditure per acre (log) | 0.189*** | 0.194 | 0.201 | 0.203 | 0.207 |
| Crop productivity (log) | 0.170*** | 0.176 | 0.182 | 0.185 | 0.189 |
| Crop commercialization | 0.052*** | 0.036 | 0.020 | 0.012 | 0.001 |
| Crop income (log) | 0.200** | 0.110 | 0.012 | -0.030 | -0.100 |

Notes: This table presents treatment effect bounds for all households using Oster (2019). The results correspond to OLS estimates with the inclusion of household controls, WTP to control for unobserved heterogeneity, and village fixed effects. The baseline coefficients in column (1) are taken from Table S9. The bias-adjusted coefficient is calculated using R_max_ equal to 1.3 times R^2^. In column (2), we assume that selection on unobservables is smaller than selection on observables (δ<1). In column (3), we assume that selection on unobservables is equal to selection on observables (δ=1). In columns (4) and (5), we assume that selection on unobservables is more (20% and 50%) than selection on observables (δ>1).
