# Supplementary material for: Personalized digital extension services and agricultural performance: Evidence from smallholder farmers in India
Source: PLoS One. 2021 Oct 28;16(10):e0259319. doi: 10.1371/journal.pone.0259319 (PMC8553076; doi:10.1371/journal.pone.0259319)
Supplement: S12 Table — (DOCX) [file pone.0259319.s014.docx]

**Table S12: L1 measure of imbalance before and after coarsened exact matching**

|  | **Before matching** | **After matching** |
| --- | --- | --- |
|  | **L1** | **L1** |
| Household head age [20-40 years] (1=Yes) | 0.130 | 7.4E-16 |
| Household head age [40-60 years] (1=Yes) | 0.127 | 1E-15 |
| Household head age [ above 60 years] (1=Yes) | 0.014 | 1.3E-15 |
| Small household size [<5 household members] (1=Yes) | 0.065 | 1.20E-15 |
| Male household head (1=Yes) | 0.058 | 1.20E-16 |
| Owns mobile phone (1=Yes) | 0.074 | 1.30E-15 |
| Illiterate ^a^ (1=Yes) | 0.029 | 1.20E-17 |
| Primary school ^a^ (1=Yes) | 0.064 | 1.10E-15 |
| Secondary school ^a^ l (1=Yes) | 0.039 | 1.40E-15 |
| Bachelor or Master ^a^ (1=Yes) | 0.100 | 1.30E-15 |
| Scheduled tribe (1=Yes) | 0.068 | 5.60E-16 |
| Scheduled caste (1=Yes) | 0.093 | 5.60E-16 |
| Other backward classes (1=Yes) | 0.137 | 1.20E-15 |
| General caste (1=Yes) | 0.025 | 1.00E-15 |
| Small farm (2.5-5 acres) | 0.056 | 1.10E-15 |
| Medium farm (5-10 acres) | 0.093 | 1.10E-15 |
| Large farm (>10 acres) | 0.058 | 2.10E-16 |
| Off-farm income (1=Yes) | 0.089 | 1.20E-15 |
| Multivariate L1 distance ^b^ | 0.679 | 7.27E-16 |
| Number of observations | 1,105 | 529 |

Notes: Descriptions of variables coarsened from original continuous variables are provided in square brackets.

^a^ Highest education attained by adult male members of the household. ^b^ Multivariate L1 distance is a measure of global imbalance as given by Iacus et al.( 2012). Perfect global balance (up to coarsening) is indicated by L1=0 and larger values indicate complete separation.
