# Supplementary material for: Personalized digital extension services and agricultural performance: Evidence from smallholder farmers in India
Source: PLoS One. 2021 Oct 28;16(10):e0259319. doi: 10.1371/journal.pone.0259319 (PMC8553076; doi:10.1371/journal.pone.0259319)
Supplement: S13 Table — (DOCX) [file pone.0259319.s015.docx]

**Table S13: Estimated associations based on coarsened exact matched data**

|  | **(1)** | | **(2)** | |
| --- | --- | --- | --- | --- |
| **Outcome variables** | **Coefficient** | **SE** | **Coefficient** | **SE** |
| Number of crops | 0.862** | (0.419) | 0.903** | (0.414) |
|  | [0.058] |  | [0.076] |  |
| Seed expenditure per acre (log) | 0.066 | (0.103) | 0.008 | (0.091) |
|  | [0.153] |  | [0.252] |  |
| Fertilizer expenditure per acre (log) | 0.166** | (0.072) | 0.150** | (0.067) |
|  | [0.050] |  | [0.076] |  |
| Pesticide expenditure per acre (log) | 0.222** | (0.100) | 0.145* | (0.085) |
|  | [0.050] |  | [0.076] |  |
| Total expenditure per acre (log) | 0.182** | (0.078) | 0.148** | (0.070) |
|  | [0.050] |  | [0.076] |  |
| Crop productivity (log) | 0.193*** | (0.069) | 0.175*** | (0.068) |
|  | [0.042] |  | [0.076] |  |
| Crop commercialization | 0.042 | (0.027) | 0.034 | (0.025) |
|  | [0.069] |  | [0.112] |  |
| Crop income (log) | 0.208* | (0.126) | 0.182* | (0.110) |
|  | [0.069] |  | [0.076] |  |
| Village fixed effects | No |  | Yes |  |
| Household controls ^a^ | No |  | Yes |  |

Notes: Columns (1) and (2) estimated using OLS on the coarsened exact matching data. * Significant at 10% level, ** Significant at 5% level, ***Significant at 1% level. Robust standard errors are shown in parentheses. Multiple hypotheses corrected sharpened *q*-values are presented in square brackets. ^a^ Household controls include age and sex of household head, highest level of education attained by adult male members, ownership of mobile phone, operated land area, peer group, off-farm income, and distance to input and output market. In column (2), household controls are in the original form as in Table 3 (i.e., without coarsening) to control for remaining imbalances.
